# “Not just about the pump”—providing sexual and reproductive health information to adolescents with heart disease

**DOI:** 10.1007/s00431-025-06357-5

**Published:** 2025-08-01

**Authors:** Annika Bay, Harpa Vidarsdottir, Elisabeth Jangsten, Åsa Burström

**Affiliations:** 1 Department of Nursing, Umea University, 90736 Umea, Sweden; 2 Department of Pediatric Cardiology, Astrid Lindgren Children’s Hospital, Karolinska University Hospital, Stockholm, Sweden; 3 Department for Women’s and Children’s Health, Karolinska Institutet, Stockholm, Sweden; 4 University West, Trollhättan, Sweden; 5 Department of Neurobiology, Care Science and Society, Karolinska Institutet, Stockholm, Sweden

**Keywords:** Congenital heart disease, Sexual and reproductive health, Adolescence, Information

## Abstract

More adolescents with congenital heart disease (CHD) are reaching adulthood and reproductive age. They face challenges transitioning from pediatric to adult health care and need to manage their health and developmental goals. The 2030 Agenda emphasizes a holistic approach to sexual and reproductive health rights (SRHR), aiming to provide accessible information to adolescents. Healthcare providers (HCPs) must offer appropriate information about CHD and SRHR issues. This study aimed to describe HCPs’ experiences of informing adolescents with heart disease about SRH. An explorative study with a descriptive design using semi-structured interviews. The qualitative content analysis method by Graneheim and Lundman was used to analyze the data. The interviews provided a wealth of data, revealing different opinions and experiences. The analysis resulted in four main categories: (1) organizational culture, (2) health care professionals’ perception of adolescents’ needs, (3) skills and ability are essential, and (4) parents’ influence on conversation

*Conclusion*: Discussing SRH is universally accepted among HCPs, but prioritization varies. HCPs must customize information, consider the timing and setting of the conversation. Enhanced knowledge and skills in SRH are essential, including strategies to overcome barriers and respectfully involve parents. Crucially, adolescents’ rights to SRH information must always be upheld.

**Table:** 

**What is Known:** • *There are challenges in the transition from pediatric to adult care in managing adolescents’ health and developmental goals. Health professionals need to provide appropriate information to adolescents about their sexual and reproductive health.*
**What is New:** • *This information needs to be tailored to the time and setting of the counselling session. Health professionals need to know more about how to provide this information and are looking for more knowledge on the subject.*

**What is Known:**

• *There are challenges in the transition from pediatric to adult care in managing adolescents’ health and developmental goals. Health professionals need to provide appropriate information to adolescents about their sexual and reproductive health.*

**What is New:**

• *This information needs to be tailored to the time and setting of the counselling session. Health professionals need to know more about how to provide this information and are looking for more knowledge on the subject.*

## Introduction

The number of adolescents living with congenital heart disease (CHD) is increasing due to improvements in diagnosis as well as surgical and medical treatment [1]. These improvements in medical development have led to an increasing number of young patients with complex care needs reaching adulthood and reproductive age [2]. For health care, this means that patients’ care needs to change focus from survival to a broader perspective. Adolescents (ages 10–24 years [3] with congenital and acquired chronic heart conditions need to be prepared for the shift of care from a pediatric health setting to an adult health setting [4, 5]. Adolescents living with complex CHD encounter distinct challenges as they transition toward greater autonomy in managing their health while simultaneously striving to establish personal and developmental life goals [4, 6]. Ensuring adolescents grow up healthy in supportive environments is a global goal. They require reliable information, accessible health services, encouraging surroundings, and opportunities to develop skills and engage in decision-making [7]. To enable people to take control of their bodies without the stigma and discrimination associated with sexual and reproductive health, information should be accessible to all [7, 8].

The 2030 Agenda for Sustainable Development, adopted by the UN General Assembly in 2015, underscores the need for integrated sexual and reproductive health and rights within the national strategies to achieve sustainable development [8]. In the Swedish school context, age-appropriate education on sexual and reproductive health and rights is described in the curriculum from preschool through upper secondary school (ages from 3 to 19 years old) [9, 10]. Schools are responsible for ensuring that all students receive education on all aspects of SRHR, including the physiological, emotional, psychological, and social well-being in relation to sexuality and reproduction [11]. The education and information are provided by a teacher and a school nurse [9, 11, 12]. However, in the context of adolescents with medical or chronic conditions, physicians and nurses play a pivotal role in giving information about SRH, as it is described that adolescents place significant trust in their knowledge and understanding of adolescents’ medical history and health-related issues [13–15]. To achieve knowledge and understanding, pediatric cardiologists and pediatric nurses, hereinafter referred to as healthcare providers (HCP), need to provide developmentally appropriate information about the CHD, health issues which include sexual and reproductive health (SRH) [4, 10, 16]. Despite guideline recommendations, many young women transfer to adulthood and adult care without adequate knowledge about SRH [17, 18]. Conversations about SRH are sparse, and the topic is often not addressed [18–20]. This can have consequences, such as the use of inappropriate contraception (medical risks or inadequate protection) and concerns and worries about pregnancy, childbirth, and transmission of their disease to future children [10, 20–22]. HCP should recognize that adolescents are interested in learning more about sexual and reproductive health (SRH) in the context of their heart disease [10, 20, 21, 23] and that they are in pivotal positions to provide information on this. Indeed, women with CHD regard their cardiologist as the principal source of information, owing to the cardiologist’s comprehensive understanding of their medical history [14].

The HCP’s expertise concerning the adolescent’s medical background means the adolescents often have great confidence in their knowledge and advice. As SRH is a topic that may be difficult to talk about, both for HCPs and adolescents, it is important to explore how the HCPs can tailor the information so that it benefits adolescents and increases their knowledge. It is nevertheless important to consider what difficulties might arise and what can facilitate the HCPs in providing that information. Hence, the aim of this study is to describe healthcare professionals’ experience in informing adolescents with heart disease about sexual and reproductive health.

## Material and methods

### Study design and setting

This is an explorative study with a descriptive design conducted using semi-structured interviews. To gain a deeper insight into the reasoning of healthcare professionals, cardiologists and nurses from seven university hospitals across Sweden were included in the study.

### Participants

The participants were consecutively recruited from seven tertiary centers in Sweden. The clinical manager of each clinic was asked to suggest participants (1–2 nurses and 1–2 pediatric cardiologists) who fulfilled the inclusion criteria. The inclusion criteria were to be employed > 2 years as a nurse or a physician at a pediatric clinic caring for adolescents with CHD or heart disease (arrythmias or cardiomyopathies). All participants suggested by the head of the clinic were handed over the study information along with a consent form in a pre-stamped envelope.

In total, 20 participants gave their written consent to participate in the study. Due to late dropouts and nonresponses, 16 HCPs participated (Table 1).

**Table 1:** Participant characteristics

Sex (*n*)	
Female	15
Male	1
Age (years, mean (± SD))	49.3 (10.2)
Profession (*n*)	
Pediatric Cardiologist	6
Nurse	10
Work experience (years, mean (± SD))	15.7 (4.2)

### Data collection

Ethical approval was provided by the Swedish Ethical Review Authority (ref 2022–05042-1, 2024–00947-02) and the study adhered to the principles of the Declaration of Helsinki throughout the research process [24].

The interviews followed an interview guide (Table 2), derived from the study aim, the existing literature, and discussion within the research group. Follow-up questions were asked for clarification. As the interviews were conducted, the data became repetitive, and no new information emerged [25]. Nevertheless, the planned interviews were conducted to ensure that the results were robust. The interviews were conducted digitally, recorded, and lasted 30–60 min. The interviews were conducted by ÅB, AB, EJ, or MB from March 2023 to June 2023 and transcribed verbatim.

**Table 2:** Interview guide

Characteristics
Age
Sex
Profession
Work experience
Experience of providing information
Describe your experiences of informing young people with congenital heart defects or heart disease about reproductive and sexual health.
If you could decide, who should provide the information, and in what way?
Perception of giving information
What information should young people with congenital heart defects or heart disease receive about reproductive and sexual health?
Should any diagnostic group be prioritized
At what age should information be given to young people?
Is it necessary to ensure that everyone receives information about sexual and reproductive health?
Obstacles and facilitating factors
(Do you feel there are obstacles/difficulties in providing information? )
Additional question
Is there anything we haven’t covered?

### Data analysis

A qualitative content analysis method [26] was selected to explore the experiences of giving information concerning sexual and reproductive health to adolescents with a CHD or other heart disease. Initially, the co-authors (ÅB, AB, EJ, and HV) read through the interviews to identify text corresponding to the aim of the study. Significant quotations were then condensed into meaning units and codes, describing the content (ÅB, AB, EJ, and HV). The next step was to group the codes into subcategories that addressed the aim. The co-authors reflected together on all the steps of the analysis and discussed differences and similarities that constitute the manifest content, which is a way to address dependability [26]. Finally, the categories were examined to find an underlying meaning, a latent content.

## Results

The interviews provided a wealth of data, offering an insight into the different opinions and experiences. The analysis resulted in four main categories: (1) organizational culture, (2) health care professionals’ perception of adolescents’ needs, (3) skills and ability are essential, and (4) parents’ influence on conversation (Table 3).

**Table 3:** Overview of categories and subcategories

Categories	Subcategories
Organizational culture	*Competence and expertise of health care professionals *
	*A trustworthy environment facilitates*
Health care professionals’ perception of adolescents’ needs	*Appropriate information and conversation*
	*Reliable relationships are important*
Skills and ability are essential	*Confident in addressing the topic*
	*Challenges in addressing the topic*
Parents’ influence on conversation	*Parents initiative facilitates*
	*Barriers of conversations*

### Organizational culture

The main category describes how the culture of an organization can facilitate information and includes two subcategories. *Competence andexpertise of health care professionals* describes the need for HCP to be skilled on the topic*. A trustworthy environment facilitates* describes that a safe environment facilitates conversation.

### Competence and expertise of health care professionals

Participants in the study indicated that it can be more challenging to initiate conversations about SRH with males than with females. Some participants described being unaware of males’ need. Some relied on the school nurses to provide SRH for males or did not consider other potential questions arising. Others experienced that males had worries about their heart condition and whether sexual activity could affect their heart.

And then I should say that the starting point is, of course, that all adolescents can have sex. That is, even those with implanted defibrillators and all sorts of things. So,what I am aiming for is that they should feel secure enough to dare to have sex (P1).

Some participants expressed that conversations with females were often facilitated by discussion about menstruation and contraception, as well as medications that may be toxic or teratogenic to a fetus. Regarding contraception and menstruation-related questions, adolescents were often referred to a youth clinic, which in turn consulted with a cardiologist to determine suitable contraception, considering the specific heart condition and other medications. It is perceived positively to have effective collaboration with adult congenital heart disease clinics (ACHD) and youth clinics, and it would be beneficial to have a contact person at those clinics interested in this patient group.

Possible ways to improve information in general were suggested, e.g., preferably in groups, as it could be easier and less personal for adolescents to discuss the subject. This information could be provided in collaboration with other professionals such as midwives at youth clinics.

### A trustworthy environment facilitates

The participants agreed that conversations about SRH should be conducted with sensitivity; there must be trust between adolescents and the HCP, and the timing should be appropriate to the maturity of the young person. It was also highlighted that it was not important whether a cardiologist or nurse provided the information; rather, it was more about possibility, opportunity, and competence. It was moreover emphasized that the conversation should take place in a more private environment, i.e., fewer people in the room and there is no risk of disturbances. However, several participants described the difficulties in finding these opportunities due to organizational factors.

Yes, but listen, we are not really so good at talking about sexual health, should we have a little workshop and discuss how we can do it? (P2).

Some HCPs expressed a desire for a checklist to recall important topics in SRH and specify (clearer) information. Some of the participants had even undergone an education in using conversation tools to use in consultation with adolescents, which included a conversation structure and assured confidentiality.

### Health care professionals’ perception of adolescents’ needs’

The main category illuminates the importance of customizing the information provided and the relevance of a relationship between HCP and adolescents, described in two subcategories: *Appropriate information and conversation* and *Reliable relationship are important*.

### Appropriate information and conversation

The HCPs described conversations about SRH with their patients. While some participants could not recall informing them about these issues, others stated that it was an area (or subject) needing improvement, insisting it was mandatory to initiate a conversation to give adolescents an opportunity to ask their questions. Some participants also argued that adolescents with corrected heart defects and a normal heart function had fewer needs and less desire for information about SRH, asking fewer questions.

They are worried, at least that’s what I believe, that they are not normal. Because if there’s something wrong with the heart, there might be something wrong elsewhere too (P3).

Some HCPs moreover expressed the need to provide tailored information based on adolescents’ medical history, even though schools also provided information about sexual and reproductive health. It was also described that it could be important to normalize and reassure adolescents that sexual activity does not harm heart function. One participant said:

It is indeed so uncommon that an adolescent has such poor heart function that it would be a problem to have sex (P4).

Several participants emphasized that, although information about sexual health and reproduction was important, the focus during medical consultations was heart disease.

### Reliable relationships are important

To enable conversations about sensitive issues, it was described as essential to have a trusting and respectful relationship between the HCP and the adolescent. The importance of confidentiality, e.g., assuring that the content of the conversation would not be shared with their parents, is also emphasized. But if desired, the HCP could assist them in informing their parents about SRH.

It was mentioned that initiating a conversation about SRH could be difficult, and important aspects were timing and a safe environment. Some felt that it was possible to talk about SRH during the ultrasound examination as it could be less sensitive when not looking each other in the eyes, but it was also noted that the patient could be in a vulnerable position as they are lying bare-chested on an examination table.

Having the parent in the ECG room and the patient alone in the ultrasound room, where the patient is undressed with a bare chest, and then… If you imagine talking to someone you don’t know while being bare-chested… (P5).

However, it was emphasized that the privacy of these conversations must be considered. In most clinics, adolescents were encouraged to meet HCP alone, but they could choose to bring their parents if they wanted. In several clinics, HCPs have undergone special education focused on preparing adolescents for transfer to adult care. The participants described conversation tools that helped to address various aspects of adolescent life in a respectful way, and some of the participants described how they tried to support adolescents to develop skills to formulate questions about their health.

### Skills and ability are essential

The main category describes the participants’ own perception of their competence and how comfortable they were in raising the topic and what challenges there were. This category contained two subcategories: *confident in addressing the topic *and *challenges in addressing the topic*.

### Confident in addressing the topic

Some participants expressed how they tried to normalize the topic to invite and initiate conversations about SRH. Sometimes, the conversation started with questions about whether they had a partner, and they were then informed that this is something they discussed with all adolescent patients at the clinic; they believe it is important for everyone, regardless of the severity of their heart condition, to receive information and feel secure in having sexual relationships. Participants emphasized the significance of raising the topic but also respecting adolescents’ readiness to discuss.

I have never felt, when leaving such a conversation, that it did not go well. The person talked about what they wanted and were ready for at that moment (P6).

### Challenges in addressing the topic

It was a challenge to assess which issue was currently relevant to discuss or inform the patients about potential risks that could occur, as it could cause the individual to limit themselves prematurely. A few questioned their responsibility to inform SRH since patients were there for heart check-ups, and the youth clinic should handle the SRH subject. Others emphasized the importance of seeing the whole person and not just focusing on physical health.

Somatic care, like cardiology, is very strict. At least in pediatric care, it’s all about how the heart functions as a pump. They forget that there is a person around the pump (P1).

An additional difficulty emerged when the youths also had an intellectual disability, adding another level of consideration. However, it was considered important that these patients receive information as they can also be sexually active and should not expose themselves to risks. Ethnicity can also be a complicated factor, especially if there is a language barrier and an interpreter is needed.

I think about language barriers. Even with an interpreter, it feels like… It doesn’t feel the same… I wonder if the interpreter is saying what I said? (P6).

Participants also express a lack of knowledge about lesbian, gay, bisexual, transgender, queer, or questioning (LGBTQ) issues and feel they need more education on the subject.

Regarding LGBTQ issues, I am not very educated. If such a case or issue arises with adolescents, I feel a bit insecure (P7).

The interviews revealed that some HCPs felt a lack of competence and knowledge on the subject and wished for more training and education on how to approach it. Addressing the subject naturally to make it less dramatic and comfortable to discuss is desired. This training can come from external sources or be something the HCPs can delve into.

### Parents’ influence on conversation

The main category describes that parents’ involvement during the visit may both facilitate and also in some cases hinder as described in two subcategories: *Parents initiative facilitates* and *Barriers of conversations*.

### Parents initiative facilitates

It was considered normal that the parents had protective instincts for their child with heart disease. Parents were often the ones discussing adolescents’ health with the HCP. It was also noted that information about SRH was often initiated by parents asking questions, typically concerning issues with menstruation.

To enable a private conversation about SRH, many adolescents preferred to be invited to the appointment alone. However, it was important not to exclude the parents. Some participants said that private conversations with adolescents would be easier if the parents were informed beforehand, and it appeared that some HCPs had received calls from parents expressing gratitude that their adolescent had been offered a private conversation about sensitive matters.

### Barriers of conversations

A barrier to sensitive and private conversation was the presence of parents during consultations. Most participants felt that parents should not be present during private conversations. However, it was noted that sometimes adolescents did not want their parents to leave, something that often depended on their level of maturity. Some participants addressed this by asking the adolescents if they accepted the presence of their parents when more private questions about health were discussed, and if the parent was present, the topic was discussed in a more general manner. There were also instances where parents did not want to leave the room, which could be avoided by assuring parents that they could discuss the results of heart examinations afterwards, as this was the focus of the medical appointment.

## Discussion

The present study demonstrates the diversity of HCPs’ experiences and views regarding informing adolescents with CHD, arrythmia, and cardiomyopathies about SRH. The study shows that, in addition to the organization of health services, the skills and expertise of health professionals and the establishment of a trustworthy environment for discussing sexual and reproductive health (SRH) with adolescents are also of great importance. This is in line with the results of Frederick et al. [27] who reported barriers such as lack of knowledge or experience and lack of resources or referrals as well as low priority of the conversation, the presence of parents or other family members, and discomfort of the patient [27]. In our study, HCPs’ perceptions of adolescents’ needs vary, with some suggesting that those with corrected heart defects require less information about SRH. However, the result stresses the importance of normalizing SRH discussions and reassuring adolescents that sexual activity does not harm heart function. Most participants addressed SRH in their consultations with the adolescents at some point, but it was more often discussed with females. It was described as easier to inform females about medication that could be toxic or teratogenic to a fetus, but many described difficulties opening the discussion on SRH with males. This difficulty might stem from a lack of understanding of males’ needs and the assumption that school nurses or midwives at youth clinics will address these issues. In a Swedish context, adolescents have the possibility to receive general information about SRHR at school, but a more specific information related to adolescent health issues can only be given in a health care setting by the HCP with specific knowledge about the patient [10, 16]. That could, in some cases, best be performed with a closer collaboration between different health care organizations, which is vital to secure appropriate information. Furthermore, it should be noted that this topic can be a challenge in other contexts as well [12, 16].

A collaboration that was highlighted by the participants was that between youth clinics and gynecologists. Issues about contraceptives and menstruation are often referred to a youth clinic or a gynecologist, who on the other hand consults a cardiologist regarding the adolescent’s heart condition [28]. Collaboration aids in giving comprehensive and specific care for adolescents when needed [29].

The study revealed a shared recognition of the importance of addressing SRH with patients; however, identifying an appropriate moment to initiate the conversation remained a significant challenge. The study shows a common agreement that SRH is an important topic for patients, but it was a challenge to find the right time to hold the conversation. Contributing factors included previous experiences, motivation, time constraints, comfort level in discussing feelings, as well as competence and knowledge related to both SRH and medical issues [12, 27, 30]. In this study, the responsibility for addressing SRH did not lie exclusively with the cardiologists; rather, it was a shared responsibility with the nurses, grounded in collaborative practice and guided by both opportunity and professional competence. However, even if SRH was acknowledged as an important issue, it was emphasized that the medical examination of the heart was prioritized. This prioritization reflects a pattern observed across various healthcare settings in the management of chronic illnesses. Similar results have been reported regarding other medical conditions [27, 30].

Also, although the topic was considered important, some of the participants had never discussed SRH with their patients. This was mainly reported among nurses in a current study, although they frequently have the opportunity and competence to give this information [31]. The aim was not to explore differences between pediatric cardiologists and nurses’ experiences, but it seemed to be more common for the pediatric cardiologists to address the topic even if several nurses were educated and experienced and had consultation with their patients.

The HCPs used different strategies when addressing the topic in a sensitive way. Sometimes, the conversation was held after the consultation and sometimes during the medical examination. However, the HCPs found it important to consider the vulnerability of the patient. It is important to maintain a youth-friendly, [32] honest, and non-judgmental approach in the consultations [32].

The HCPs called for education about medical content, knowledge about SRH and conversation tools, and several of the HCPs described how conversation tools were used as an aid in addressing the topic [33]. The participants in the study acknowledged adolescents’ rights for information about SRH, and many of the participants tried to provide SRH information in an appropriate way.

The results of this study suggest that HCP aim to provide adequate information, but they encounter both obstacles and facilitating factors. The importance of seeing the whole person, rather than just focusing on physical health, is emphasized. Language barriers and a lack of knowledge about LGBTQ issues are identified as additional challenges that need to be addressed through further education and training. Another challenge may be to inform adolescents with intellectual disabilities about SRH. This requires extra consideration and customization of the information, for example by using a specific pedagogical framework for properly tailored information [34].

Parents play a significant role in SRH conversations, often initiating discussions about their child’s health [35, 36]. While private conversations with adolescents are preferred, it is important not to exclude parents entirely as it can be difficult for an adolescent to have private conversations without parents for support [37]. Strategies to manage parents’ presence during consultations include reassuring them that they can discuss the results of heart examinations afterward. However, it is important to recognize the difference between general information and private information for individual adolescents [36].

### Methodological considerations

A qualitative design with individual interviews was chosen to gain deeper and broader insight into the perspectives of key health influences to understand factors affecting conversation and information about sexual and reproductive health. To measure trustworthiness and to achieve credibility and transferability in the current study, the recruitment of participants was designed to find those who had experience in caring for adolescents with CHD [29]. It was seen as a strength that experienced pediatric cardiologists and nurses from seven hospitals in Sweden shared their view regarding the issue, and they also expressed that it was positive to be interviewed about the topic. Several limitations need to be addressed. Participants reported that it was generally easier to initiate conversations with female adolescents, particularly when discussing topics such as menstruation. This perceived ease of communication may, however, be influenced by the predominance of female participants in the study, suggesting that shared gender identity could facilitate the identification of common experiences and concerns. The limited sample size may restrict transferability; however, detailed descriptions of participant characteristics, data collection, and analysis process enhance transparency. Presentation of the results, together with appropriate citations, strengthens credibility and increases the possibility of transferability by allowing the reader to find alternative interpretations [38]. Then there are additional factors, such as social factors, that affect providing information about SRH [39]. These need to be further explored in health care settings that care for adolescents with heart disease.

## Conclusion

Discussing SRH is universally accepted among HCPs, yet prioritization varies. HCPs must customize SRH information by considering the timing and setting of the conversation. Enhanced knowledge and skills in SRH are essential, including strategies to overcome barriers and respectfully involve parents. Crucially, adolescents’ rights to SRH information must always be upheld. Some questions for further research include when it is the proper time to inform, what information adolescents with congenital and acquired chronic heart conditions need and desire, the context in which it should be delivered, and the most effective methods for communication.

## Data Availability

No datasets were generated or analysed during the current study.

## Abbreviations

CHD: Congenital heart disease
HCP: Healthcare providers
P: Participant
SRH: Sexual and reproductive health
SRHR: Sexual and reproductive health rights
